# Development of internal standard for lipoprotein subclass analysis using dual detection gel-permeation high-performance liquid chromatography system

**DOI:** 10.1042/BSR20220291

**Published:** 2022-06-01

**Authors:** Mei Ogino, Takahiro Kameda, Yume Mutsuda, Hideko Tanaka, Junichiro Takahashi, Mitsuyo Okazaki, Masumi Ai, Ryunosuke Ohkawa

**Affiliations:** 1 Analytical Laboratory Chemistry, Graduate School of Medical and Dental Sciences, Tokyo Medical and Dental University (TMDU), 1-5-45 Yushima, Bunkyo-ku, Tokyo 113-8519, Japan; 2 Immuno-Biological Laboratories Co., Ltd. 1091-1 Naka Aza-Higashida, Fujioka-shi, Gunma 375-0005, Japan; 3 TMDU, 1-5-45 Yushima, Bunkyo-ku, Tokyo 113-8519, Japan; 4 Insured Medical Care Management, Graduate School of Medical and Dental Sciences, TMDU, 1-5-45 Yushima, Bunkyo-ku, Tokyo 113-8519, Japan

**Keywords:** gel-permeation high-performance liquid chromatography, internal standard, lipoprotein profile, lipoprotein subclasses, quinone pigment, Trinder reaction

## Abstract

The LipoSEARCH® System is an innovative lipoprotein class analysis method based on gel-permeation high-performance liquid chromatography (HPLC). This system uses a gel permeation column to separate the major lipoprotein subclasses (chylomicron, very low-density lipoprotein, low-density lipoprotein, and high-density lipoprotein) in serum according to particle size and splits them into two pathways to measure total cholesterol (TC; esterified + unesterified cholesterol) and triglyceride (TG) concentrations simultaneously to obtain chromatograms for each.

These chromatograms were analyzed based on the results of the calibration serum by fitting Gaussian curves to profile the 20 lipoprotein subclasses defined in detail. An important assumption of this HPLC system is its simultaneous detection of two pathways to guarantee the accuracy of each analysis. Therefore, in the present study, we investigated the development of an internal standard that can guarantee the simultaneous detection of this system by adding a pigment to the serum. We focused on quinone pigments with absorption at 550 nm, which is the wavelength used for the enzymatic assay of TC and TG concentrations in the system. As a result, we succeeded in producing overlapping pigment peaks that appeared after the analytical chromatograms in two pathways. It is also suggested that the pigment solution as an internal standard is stable in freezing storage and has little effect on the analysis. The developed internal standard is expected to contribute to the accuracy assurance of lipoprotein analysis by this dual-detection HPLC system.

## Introduction

The relationship between lipoprotein profiles and cardiovascular disease (CVD) has been studied for a long time. In particular, high-density lipoprotein (HDL) and low-density lipoprotein (LDL) are involved in cholesterol metabolism as carriers between the liver and peripheral tissues, and the measurement of their serum cholesterol levels (HDL-C and LDL-C, respectively) has been widely accepted to predict the risk of CVD [1,2]. In addition, triglyceride (TG), which is carried mainly in chylomicrons (CM) and very low-density lipoprotein (VLDL), is also known to be related to atherogenicity, and its serum levels have been evaluated. However, the estimation of serum HDL-C, LDL-C, and TG concentrations does not always cover the various types of patients with CVD as risk factors because even particles that are classified as the same major classes (CM, VLDL, LDL, and HDL) are heterogeneous in terms of size and function, and quantitative evaluations of these four lipoprotein classes by measuring their total cholesterol (TC; esterified + unesterified cholesterol) and TG levels are not sufficient to understand their lipoprotein metabolism.

For example, small-dense LDL is the most atherogenic lipoprotein LDL subclass compared with other LDL subclasses [3]. Cholesterol efflux capacity, an important function of HDL for atheroprotection, has also been reported to vary by HDL subclass classified based on its particle size [4]. Thus, detailed lipoprotein subclass analysis is of great importance. The ultracentrifugation technique has been widely used as a gold standard to separate and analyze lipoprotein fractions from serum. However, it takes a lot of time for separation, and a large amount of serum is required to obtain sufficient isolated samples [5,6].

One method has shed light on this problem. The LipoSEARCH® system, gel-permeation high-performance liquid chromatography (GP-HPLC) system with on-line dual detection of TC and TG in the lipoprotein subclasses, allowed for a more detailed analysis [7–10]. Using this method, by injecting a small volume of serum into a GP-HPLC apparatus, the TC and TG concentrations and the corresponding number of particles of the four major classes (CM, VLDL, LDL, and HDL) and 20 detailed subclasses of lipoproteins were estimated [8]. To analyze the lipoprotein profile obtained from this system, the matching of the outputs of the two detectors for measuring TC and TG is indispensable. However, equipment failure based on time- and usage-related deteriorations can occur, resulting in the production of a random error, and the judgment of the TC and TG dual detection by apparent profiles for each patient is difficult. Therefore, the present study aimed to develop an internal standard (IS) that can guarantee the simultaneous measurement of TC and TG levels using the GP-HPLC method.

The principle of TC and TG measurement by enzymatic reaction is that hydrogen peroxide as a reaction product causes the Trinder reaction to produce a quinone pigment with absorption at 550 nm [8]. By using a pigment solution with absorption at 550 nm, which is the same wavelength used for this system, the peaks of the pigments overlapping with those of the two detection systems could be obtained in addition to the serum chromatograms. Based on this hypothesis, we attempted to improve the measurement accuracy of this GP-HPLC method by developing an IS.

## Methods

### Reagents

Trinder reagents, including N-ethyl-N-3-sulfopropyl)-3-methoxyaniline (ADPS), N-ethyl-N-(3-sulfopropyl)aniline (ALPS), N-ethyl-N-(2-hydroxy-3-sulfopropyl)-3,5-dimethoxyaniline (DAOS), N-(2-Hydroxy-3-sulfopropyl)-3,5-dimethoxyaniline (HDAOS), 3-(N-Ethyl-3-methylanilino)-2-hydroxypropanesulfonic acid (TOOS), and 3-(N-Ethyl-3-methylanilino) propanesulfonic acid (TOPS) were obtained from Dojindo Co. (Kumamoto, Japan). Horseradish peroxidase (HRP) and 4-aminoantipyrine (4-AA) were obtained from Wako Co. (Osaka, Japan).

All reagents prepared for IS were dissolved in an HPLC running buffer before use. HPLC running buffer, reagents for TC and TG detection were prepared according to a previous study [8], with minor modifications.

### Serum samples

Normal serum samples were obtained from healthy volunteers who provided written informed consent at Tokyo Medical and Dental University. The present study was approved by our institutional research ethics committee (M2015-546). The present study was performed at Tokyo Medical and Dental University.

### Preparation of quinone pigment as IS

Six Trinder reagents, HDAOS, DAOS, ADPS, ALPS, TOOS, and TOPS, were selected based on the molecular weight and maximum absorption wavelength as quinone pigments proposed for IS. Using these Trinder reagents, six types of reagent A were prepared with 3 mmol/L each Trinder reagent and 10 kU/L HRP as the final concentration. In addition, 20 mmol/L 4-AA solution was prepared as reagent B. Reagents A and B were mixed at a ratio of 3:1, and H_2_O_2_ was added to a final concentration of 5 mmol/L to produce each derived quinone pigment (dHDAOS, dDAOS, dADPS, dALPS, dTOOS, and dTOPS) by Trinder reaction. The composition of the reagents was based on a previous study using quinone dyes [11].

### Analysis using GP-HPLC dual detection system

Lipoprotein subclass analysis by GP-HPLC was conducted using the LipoSEARCH® system as described previously [8]. The system consisted of a Shimadzu Prominence HPLC system (Shimadzu Inc., Japan) equipped with four LC-20AD pumps, a CTO-20A column oven, an SPD-20A ultraviolet detector, an SIL-30AC auto injector, and a CBM-20A system controller. Eight microliters of samples injected into the GP-HPLC system passed through the tandemly connected SkylightPakLP1-AA gel permeation columns (Immuno-Biological Laboratories Co., Ltd., Gunma, Japan, 300 mm x 4.6 mm I.D.) maintained at 37°C, and then were equally split into two paths. Each split effluent was mixed with TC and TG reagents to allow the enzymatic reaction at 37°C. Absorbance units were measured simultaneously at 550 nm using a dual-detection system. The flow rates of the running buffer and reaction regents were 0.24 and 0.12 mL/min, respectively.

### Calculation of TC and TG concentration lipoprotein subclasses

The obtained chromatograms were analyzed using the LipoSEARCH® system [8]. TC and TG values of each lipoprotein subclass were calculated by measuring the calibration serum and fitting a Gaussian curve to the chromatograms.

### Quinone pigment selection as IS

Quinone pigments were prepared from each of the six Trinder reagents and analyzed using a GP-HPLC system. A two-fold diluted IS solution with running buffer was injected and analyzed in the same flow as the serum samples. Pigment peaks were detected to confirm their peak shapes and retention times.

### IS addition to serum samples

Serum samples, IS solution, and running buffer were mixed in a 2:1:1 ratio. The TC and TG values of 20 lipoprotein subclasses were compared to the two-time dilution of serum with buffer only to verify the effect of the addition of IS.

### Storage stability of the quinone pigment as IS

To investigate the stability of the internal solution, the pigment solution prepared from ADPS was stored at room temperature (22–24°C) or −80°C for 10 days. The solution was added to the serum as IS, and the pigment peakr attenuation at 550 nm by storage was confirmed by HPLC.

### Verification of the efficacy of IS addition

The flow rate setting of the reaction reagent is usually 0.12 mL/min as mentioned before. The flow rate of one channel was intentionally changed to 0.10 mL/min for TC and to 0.14 mL/min for TG as a reproduction of an accidental error.

### Statistical analysis

Comparisons of TC or TG values with and without IS were performed using paired  *t*-tests. Statistical significance was set at *P*<0.05.

## Results

### Quinone pigment selection for serum 20 lipoprotein subclasses analysis

**Figure 1:**
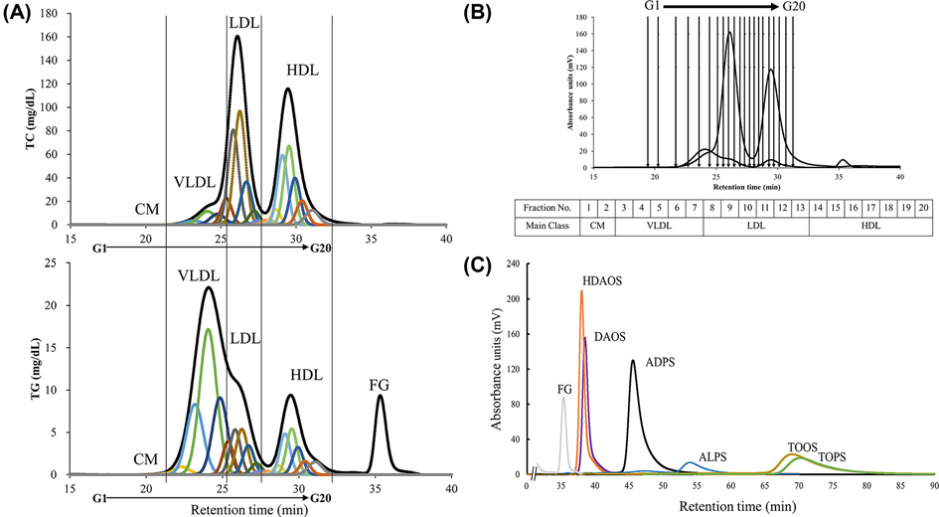
Quinone pigment selection for serum 20 lipoprotein subclasses analysis (**A**) Representative patterns for resolution of the serum four major lipoprotein classes (black line) into 20 detailed subclasses of lipoproteins (G1-G20, colored lines) using the Gaussian curve fitting technique, following the analysis for TC (A, top) and TG (A, bottom) levels by GP-HPLC system. (**B**) Correspondence of the detailed subclasses (G1-G20) to the major lipoproteins (CM, VLDL, LDL, and HDL). Black lines are drawn at the boundary time between each subclass. (**C**) Six Trinder reagent-derived quinone pigments were injected into the GP-HPLC system. Each sample was eluted with running buffer only (without TC and TG reagents) and monitored at 550 nm. The overlaid chromatogram of these pigment solutions (dHDAOS, dDAOS, dADPS, dALPS, dTOOS, and dTOPS) is representative of three separate experiments. A typical free glycerol peak pattern detected using TG reagent is also overlaid to compare its retention time (36 min) with the others.

First, healthy human serum was analyzed by the GP-HPLC system. Typical chromatograms from normal subjects were obtained as shown in previous report [8] (Figure 1A). Using the Gaussian curve fitting technique, the major four lipoprotein classes (CM, VLDL, LDL, and HDL) were resolved into 20 detailed lipoprotein subclasses as G1 and G2 (CM), G3 to G7 (VLDL), G8 to G13 (LDL), and G14 to G20 (HDL) (Figure 1A,B). To seek an appropriate quinone pigment used as IS for the serum 20 lipoprotein subclasses analysis, six types of quinone pigment solutions prepared from the Trinder reagents (3 mmol/L HDAOS, DAOS, ADPS, ALPS, TOOS, or TOPS) were injected into the GP-HPLC system. The peak retention times and shapes of the six quinone pigments were determined (Figure 1C). Sharp peaks of dHDAOS and dDAOS were observed with retention times of approximately 38 and 39 min, respectively. These pigments were eluted earlier than the others, but these peak retention times were very close to 36 min, which is the retention time of the peak of free glycerol in serum. Following this, the peaks of dADPS (46 min) and dALPS (54 min) were observed, and the slowest peaks were dTOOS (70 min) and dTOPS (71 min). The dTOOS and dTOPS showed much lower peak heights than the others, corresponding to their retention times, while the peak height of dALPS was lowest in spite of its middle retention time. Based on these results, we considered the pigment derived from ADPS to be the most suitable IS.

### The effect of IS addition on lipoprotein analysis

**Figure 2:**
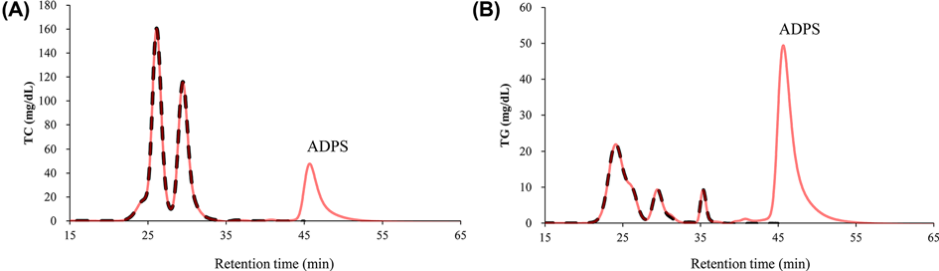
Chromatograms of serum samples with and without IS Serum samples with or without IS were injected into the GP-HPLC system, followed by reaction with TC (**A**) and TG (**B**) reagents. Comparison of chromatograms between serum alone (black, dotted line) and serum with quinone pigment from ADPS (red, solid line) as IS. The chromatograms are representative of three separate experiments.

**Figure 3:**
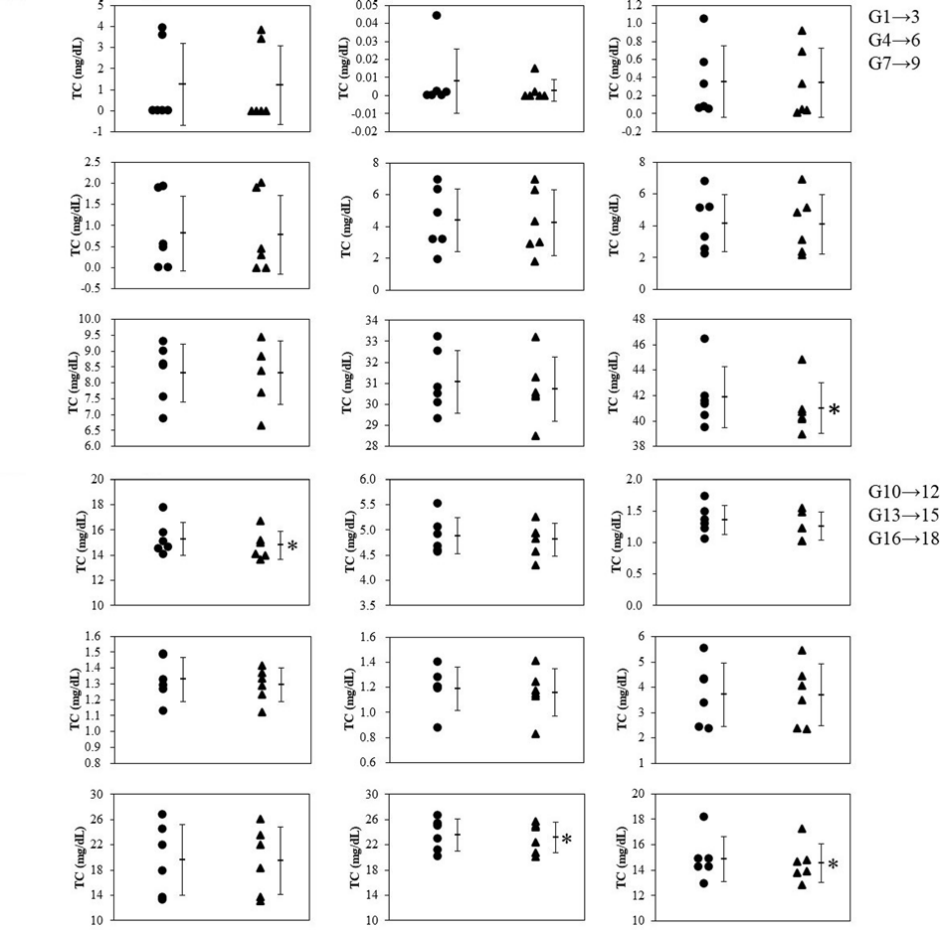
Effect of IS solution on the TC values in the G1-G18 subclasses IS solution was added to the serum samples obtained from six healthy human volunteers, and each sample was analyzed by GP-HPLC system to investigate the effect of IS on the TC values in each subclass. Dot plots show the comparison of TC values in subclasses G1-G18 between without (●) and with (▲) the IS. Each bar represents mean ± SD,* n*=6. *P<0.05, paired *t*-tests

**Figure 4:**
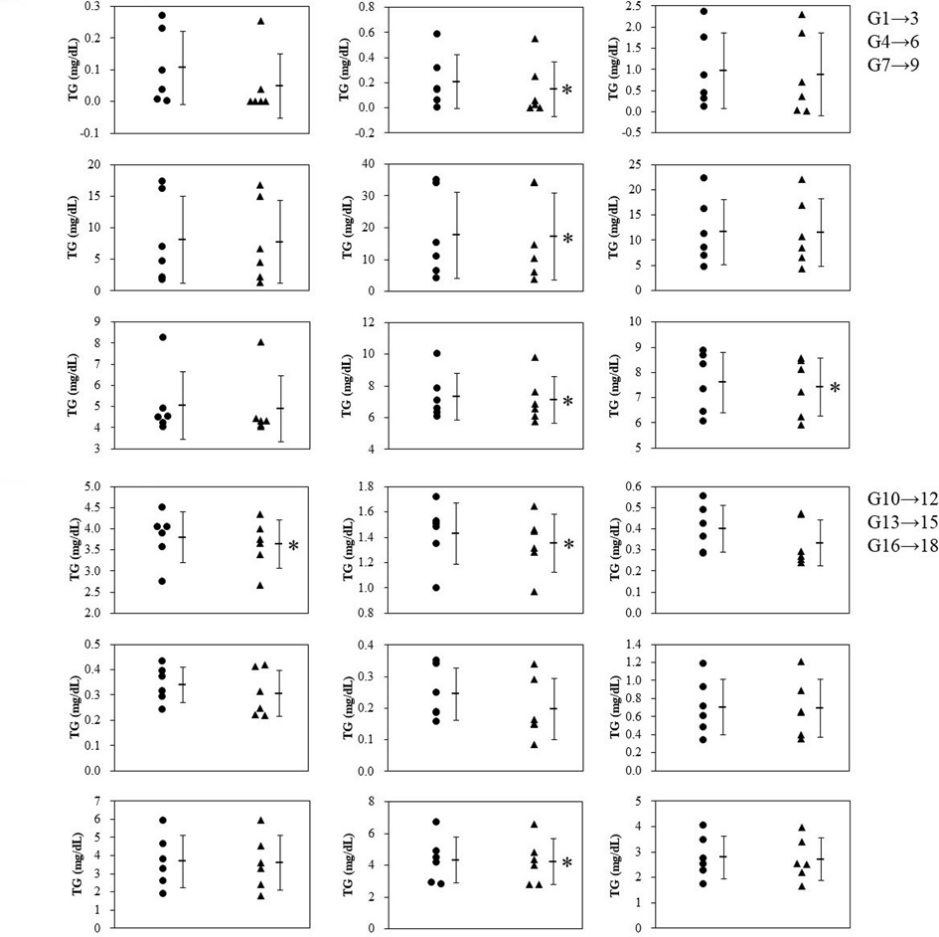
Effect of IS solution on the TG values in the G1-G18 subclasses IS solution was added to the serum samples obtained from six healthy human volunteers, and each sample was analyzed by GP-HPLC system to investigate the effect of IS on the TG values in each subclass. Dot plots show the comparison of TG values in the subclasses G1-G18 between without (●) and with (▲) the IS. Each bar represents mean ± SD, *n*=6. *P<0.05, paired *t*-tests

**Figure 5:**
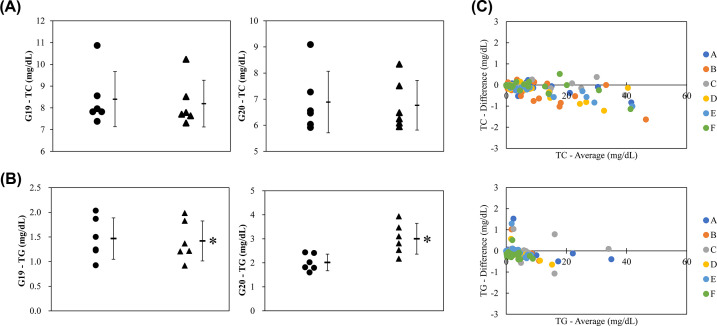
Effect of IS solution on the TC and TG values of 20 subclasses IS solution was added to the serum samples obtained from six healthy human volunteers, and each sample was analyzed by GP-HPLC system to investigate the effect of IS on the TC and TG values in each subclass. Dot plots show the comparison of TC (**A**) and TG (**B**) values in HDL subclasses G19 and G20 between without (●) and with (▲) the IS. Each bar represents mean ± SD, *n*=6. *P<0.05, paired *t*-tests. (**C**) Bland-Altman plots with and without IS from TC (top) and TG (bottom) values in all subclasses (G1 – G20).

The selected ADPS solution was added to healthy human serum, and the mixture was analyzed by GP-HPLC. Figure 2 shows representative chromatograms of the sera with and without IS. As expected, the peak of dADPS appeared at 46 min in both chromatograms of TC and TG levels (Figure 2A,B), consistent with the results shown in Figure 1. Comparing the profiles with and without the IS, both apparent profiles matched well. To further prove that the addition of IS does not affect the results of TC and TG values of lipoprotein subclasses, the Gaussian curve fitting technique using the LipoSEARCH® system was applied to the profile of serum samples with IS. Regarding to the G1 to G18, there was small but significant differences of TC and TG levels in the subclasses (4 of 18 for TC, and 9 of 18 for TG) between with and without the ADPS solution (Figures 3 and 4). However, the TG levels in G20 with IS were quite higher than those without IS whereas there was no significant difference in the TC levels (Figure 5B). With regard to the G19, TG levels with IS was significant but slight lower than without IS, and there was no significant difference in the TC levels (Figure 5A,B). Moreover, since IS might affect both lipid levels in some subclasses, including G19 and G20, we further analyzed these results by the Bland–Altman analysis showing the relationship between the magnitude of the error due to the addition of IS and the measured values; in the case of TC, there was no characteristic relationship, but the results of TG analysis showed a large positive error in the lower part of the mean value (Figure 5C). These error values (six values from the six individual shown as colored dots in Figure 5C) were related to the G20 as shown in Figure 5B.

### Repeatability of IS detection and stability against storage

**Figure 6:**
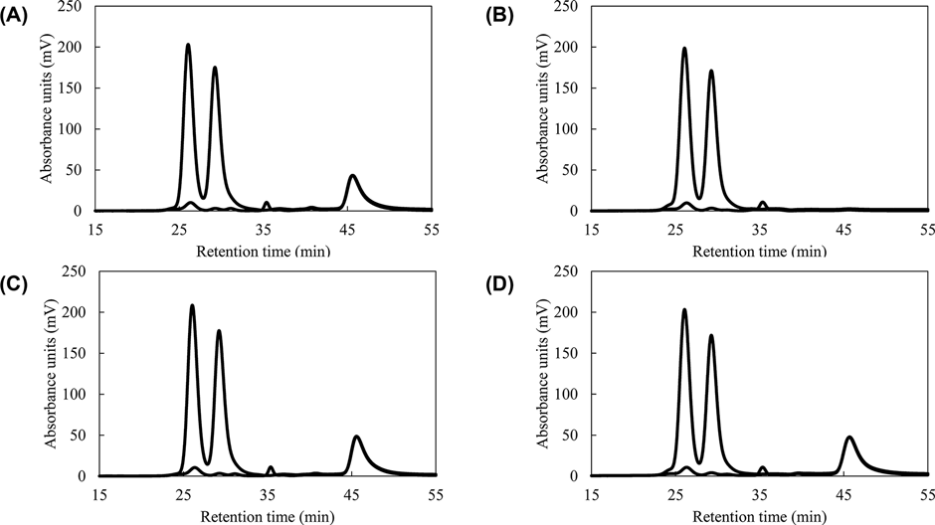
Quinone pigment stability depending on the temperature The IS solution was added to the serum. (**A**) The mixture was immediately analyzed by GP-HPLC system. (**B**) The rest of the IS solution was stored at room temperature (22–24°C) for 10 days before addition to the same serum, and the serum was analyzed. In the same manner, chromatograms of sera including IS solution, which was added immediately (**C**) and after 10 days of storage at −80°C (**D**) before analysis were compared. The chromatograms are representative of three separate experiments.

Quinone pigments gradually fade over time. According to the evaluation of the stability of the quinone pigment used in the current study, the absorbance of IS peak was measured 20 times continuously for repeatability test. The coefficient of variation was 2.9% for one detector (for TC measurement) and 3.1% for the other detector (for TG measurement). Next, we investigated IS stability against temperature. The pigment stored at room temperature (22–24°C) showed a peak reduction corresponding to a maximum of 97% after 10 days of storage (Figure 6A,B). As shown in Figure 6B, the IS peak was no longer visible after storage at room temperature (22–24°C). On the other hand, when the pigment solution was stored at −80°C, little attenuation in peak height was observed (Figure 6C,D). The IS peaks showed a decrease of up to 2.4% after storage at --80°C, which suggests that the coloration was generally comparable to that before storage.

### Verification of the efficacy of IS addition

**Figure 7:**
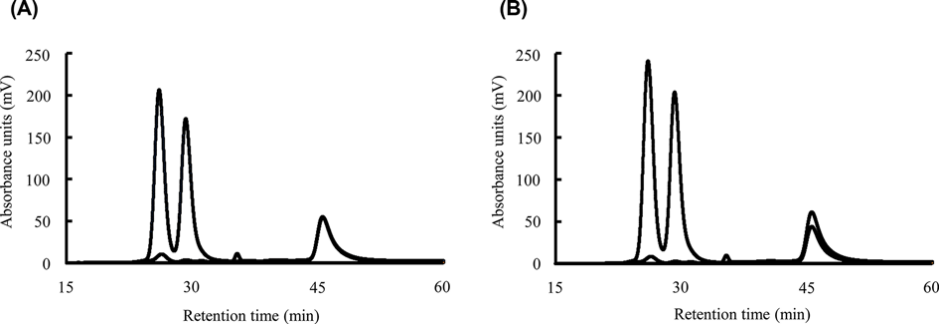
Effectiveness verification of the IS addition Serum including IS solution was analyzed by a GP-HPLC system with normal or assumed accidental conditions. (**A**) The flow rate of the two reagent was equal, 0.12 mL/min. (**B**) The flow rate of the reaction reagent was changed to 0.10 mL/min (for TC) and 0.14 mL/min (for TG). Chromatograms are representative of three separate experiments.

First, we confirmed the repeatability of the retention time of IS peak. When same IS solution was measured 20 times repeatedly, the CV of the retention time from both dual lines was less than 1%. Next, the flow rate of the pumps for the reaction reagents was deliberately shifted to reproduce the accidental error in the analysis. As shown in Figure 7A,B, both results seemed to be obtained without any problem if we focused only on the serum chromatogram (retention time of 15–30 min). This is because the TC and TG results obtained from a single analysis do not have peaks that are drawn in the same way. If we continue the calculation, there are no particular problems, and normal and incorrect results cannot be distinguished. However, because of the appearance of the peaks with the IS at 46 min, it can be noticed that the distribution of the sample is unequal, and that there is an error in the analysis. The result confirmed that the peaks of the IS appearing later in the chromatogram reflect accidental errors. Changing the flow rate of the pump created a difference of up to 7.6 mg/dL for TC and 1.6 mg/dL for TG in representative results compared with normal ones.

## Discussion

The LipoSEARCH® system is a breakthrough method that enables simultaneous analysis of TC and TG levels in the detailed lipoprotein and is very useful for basic and clinical research, that is, metabolic syndrome and diabetes [12–15]. Moreover, this HPLC system calculates not only the concentrations but also the particle counts; it is precise and highly sensitive enough to provide accurate results comparable with NMR [16].

Verification of synchronization in TC and TG chromatograms is required to further ensure the results obtained from each sample. Therefore, in the present study, we aimed to develop an IS for lipoprotein subclass analysis using a dual-detection HPLC system.

First, six types of Trinder reagents were selected to produce quinone pigments with a maximum absorption wavelength of 550 nm, which is the primary wavelength used in the LipoSEARCH® system. From the results shown in Figure 1C, ALPS, TOOS, and TOPS with slow retention times are incompatible in terms of analysis efficiency. A low peak height is also undesirable for the IS. Regarding the retention time, HDAOS was the fastest, followed by DAOS. However, they were not selected because their peaks were too close to the peak of free glycerol, which is also available as an analyte. Therefore, for the above reasons, we concluded that the quinone pigment derived from ADPS was the most suitable for IS. When serum, including the dADPS, was analyzed by HPLC, the dADPS peaks appeared simultaneously in two pathways at the latter retention time than the serum chromatogram. Therefore, the quantitative and temporal agreement of the measurements could be successfully confirmed by pigment addition to the samples as an IS.

To confirm that the addition of the pigment as an IS does not affect the TC and TG measurements, we compared the analytical results with and without the IS. When the results obtained from six subjects were analyzed, statistically significant differences in TC and TG levels were found in a total of four and nine subclasses, respectively. On the other hand, as shown in the Bland–Altman plot, there was no proportional or constant error in the measurements. Although significant differences in statistical results are scattered across several subclasses (G1–G19), the obvious variation in the paired data groups with and without IS was not observed as confirmed in Figures 3–5. Moreover, the absolute difference between the measured values with and without the addition of the IS was at most 1.63 mg/dL for TC and 1.52 mg/dL for TG. Therefore, considering that the IS will be added to every calibrator serum and sample as an analytical procedure, it is unlikely that the IS addition can make the results inaccurate. With regard to the TG levels in HDL subclass G20, however, a larger positive error was observed in G20 from serum with IS than in the other subclasses. This might occur because the pigment was bound to a certain serum protein that would be separated into G20 and measured at that retention time. As has been known that albumin is close in size and density to small HDL subclasses, it often contaminates HDL fractions isolated by ultracentrifugation [17]. Since albumin formed a complex with pigment and appeared at the same retention time as the small HDL subclass around G20, some positive differences might be observed. In particular, the corresponding small HDL subclass has a retention time that corresponds here to around G20; The reason why these false high values were estimated for only TG is probably because the scale of TG values is smaller than that of TC values, resulting in a relatively higher error in G20-TG. Same reason can be applied to the fact in G1–G19 that the number of subclasses which showed statistical differences was larger for TG than those for TC. However, if we assume that the calibrator serum and samples are measured in the same way when ISs are added uniformly, there will be no effect on the interpretation of the results. Consequently, it has been shown that the uniform addition of dye to serum as an IS does not affect the interpretation of the results obtained by this method.

Considering the use of the pigment solution as an IS for routine analysis, the stability of the IS solution after derivatization of the Trinder reagent is essential. Therefore, we investigated whether the IS solution could be stored without the need for preparation for every analysis. The quinone pigment produced by the Trinder reaction gradually faded over time. We hypothesized that the pigment solution could be stored for a long time by freezing, considering that this fading reaction depends on temperature. As a result, the frozen pigment solution retained its coloration with almost no decrease in peak height even after 10 days. Therefore, it was suggested that the pigment solution as an IS is not necessarily required to be prepared for every analysis and can be stored at −80°C for several days.

To prove that accidental errors can be detected by the presence of peaks in the IS, two pairs of pumps used for the TC and TG reagents were intentionally malfunctioned at a flow rate. The hypothesis was that the chromatograms of TC and TG would reflect the change in flow rates, and this could be detected from the deviation of the IS peak. As a result, it was possible to read that the analysis was not performed properly due to the shift in the peaks of the IS. Because the patterns of failed chromatograms are apparently similar to the successful ones, finding accidental errors in the analysis is difficult. Furthermore, this is mostly impossible in the case of abnormal chromatograms obtained from patient samples. Accidental errors can be clearly detected in the presence of IS peaks. In the present study, we observed a slight difference of 0.04 mL/min between the two pathways, and the difference in values caused by this condition was up to 7 mg/dL for TC and 2 mg/dL for TG. These results proved that the IS developed in the present study is useful to make accidental analysis errors easier to detect and prevent inaccurate data from being adopted. To the best of our knowledge, there is no precedent for such a study using pigments as IS for GP-HPLC dual detection system. A limitation of the present study is that only simulated experiment was performed for evaluation of the developed IS. Further verification is required at the time of actual analysis to prove its usefulness.

In conclusion, we newly developed an ideal IS for a GP-HPLC system using a derivative from ADPS to ensure the synchronization of dual detection. Using this IS, more reliable data would be assured for each individual analysis.

## Data Availability

The data used to support the findings of this study are available from the corresponding author (Ryunosuke Ohkawa, Graduate School of Medical and Dental Sciences, Tokyo Medical and Dental University (TMDU), ohkawa.alc@tmd.ac.jp) upon request.

## Abbreviations

CVD: cardiovascular disease
HDL: high-density lipoprotein
HPLC: high-performance liquid chromatography
IS: internal standard
LDL: low-density lipoprotein
TC: total cholesterol
VLDL: very low-density lipoprotein
